# Statement of Retraction: Statins significantly repress rotavirus replication through downregulation of cholesterol synthesis

**DOI:** 10.1080/19490976.2022.2151146

**Published:** 2022-11-23

We, the Editors and Publisher of the journal *Gut Microbes*, have retracted the following article:

Shihao Ding, Bingtiny Yu, and Anneke J. van Vuuren. Statins significantly repress rotavirus replication through downregulation of cholesterol synthesis. Gut Microbes. 2021;13(1):1955643. doi: 10.1080/19490976.2021.1955643.

An investigation undertaken by Erasmus MC – University Medical Center Rotterdam and subsequently upheld in an investigation by the Netherlands Board on Research Integrity (LOWI) have found that this paper was published without co-author consent and without appropriate acknowledgement of co-authors involved in the creation of the article. Additionally, the investigations found the data included within this article was published without appropriate authorization. Due to these reasons, the article shall be retracted.

We have been informed in our decision-making by our policy on publishing ethics and integrity and the COPE guidelines on retractions.

The retracted article will remain online to maintain the scholarly record, but it will be digitally watermarked on each page as ‘Retracted’.

